# Noise in ICUs: Review and Detailed Analysis of Long-Term SPL Monitoring in ICUs in Northern Spain

**DOI:** 10.3390/s22239038

**Published:** 2022-11-22

**Authors:** Awwab Qasim Jumaah Althahab, Branislav Vuksanovic, Mohamed Al-Mosawi, Maria Machimbarrena, Roi Arias

**Affiliations:** 1 School of Energy and Electronic Engineering, Faculty of Technology, University of Portsmouth, Portsmouth PO1 3DJ, UK; 2 Department of Electrical Engineering, College of Engineering, University of Babylon, Hillah 51001, Iraq; 3 School of Architecture, Applied Physics Department, University of Valladolid, 47014 Valladolid, Spain; 4 Proceso Digital de Audio S.L., 09001 Burgos, Spain

**Keywords:** ICUs, SPLs, noise analysis, FFT, histogram

## Abstract

Intensive care units (ICUs) are busy and noisy areas where patients and professional staff can be exposed to acoustic noise for long periods of time. In many cases, noise levels significantly exceed the levels recommended by the official health organisations. This situation can affect not only patient recovery but also professional staff, making ICUs unhealthy work and treatment environments. To introduce the measures and reduce the acoustic noise in the ICU, acoustic noise levels should first be measured and then appropriately analysed. However, in most studies dealing with this problem, measurements have been performed manually over short periods, leading to limited data being collected. They are usually followed by insufficient analysis, which in turn results in inadequate measures and noise reduction. This paper reviews recent works dealing with the problem of excessively high noise levels in ICUs and proposes a more thorough analysis of measured data both in the time and frequency domains. Applied frequency domain analysis identifies the cyclic behaviour of the measured sound pressure levels (SPLs) and detects the dominant frequency components in the SPL time series. Moreover, statistical analyses are produced to depict the patterns and SPLs to which patients in ICUs are typically exposed during their stay in the ICU. It has been shown that the acoustic environment is very similar every night, while it can vary significantly during the day or evening periods. However, during most of the observed time, recorded SPLs were significantly above the prescribed values, indicating an urgent need for their control and reduction. To effectively tackle this problem, more detailed information about the nature of noise during each of the analysed periods of the day is needed. This issue will be addressed in the continuation of this project.

## 1. Introduction and Background of Noise in ICUs

A typical ICU is equipped with medical devices used by nurses, physician specialists, and other staff to deliver care to ICU patients. Traditionally, ICUs also have pagers, alarms, beeps, monitors, and telephone systems, which, together with the conversations and activities of medical staff, visitors, and patients around the clock, can create a high amount of acoustic noise. A patient’s stay in the ICU is therefore usually accompanied by exposure to environmental noise pollution [1]. Frequent exposure to high sound levels for long periods can be very annoying and may lead to serious psychological and physiological effects.

Patients exposed to excessive SPLs can suffer from sleep disturbances, which can in turn cause fatigue during the day [2]. Sleep deprivation may also weaken cognitive functions, leading to serious complications such as delirium [3,4]. Studies have shown negative effects of noise on patients’ behaviour, such as stress, anxiety, and fatigue, which can further cause changes in blood pressure, heart rate, stimulating adrenaline, the immune response, hearing, and hormone production [5,6,7]. Christensen in [8] has investigated the physiological effects of noise on patients and revealed that exposure to high sound levels in ICUs can cause mental dysfunction. Longer hospital stays and prolonged recovery have severe consequences for patients exposed to acoustic noise during their hospitalisation. Acoustic noise in ICUs can also have a negative effect on the performance of healthcare staff. Working in noisy environments can lead to some psychological changes, such as mental stress and tension, that can cause deficient sustained memory, reduced sensitivity, impaired execution of tasks, and consequently, increase the potential for errors and wrong decisions during working days [9]. Objective measures, such as heart rate and blood pressure, can also be associated with noise change [10]. The study presented by Ryherd et al. [11] has revealed that acoustic noise could affect 91% of nurses in their routine work. It was found that the highest sound level accepted should not exceed 45 dBA for fully understood conversation between staff throughout the room. Impaired speech communication and poor concentration may increase the problems and accidents associated with making important decisions, which in turn may lead to a loss of trust from ICU patients [12].

With the progress of technology and more equipment being added to the ICU, the levels of noise have been steadily increasing through the years [13]. Thus, the reduction of noise has become one of the most acute problems in hospitals and ICUs in particular. Reducing and maintaining sound levels within the guidelines, given in Table 1, recommended by the World Health Organisation (WHO), the International Noise Council (INC), and the National Institute for Occupational Safety and Health (NIOSH), has been proven to be a very difficult task, almost impossible to achieve in many cases [1,6]. The premise of this work is that this is not a lost battle, provided an accurate recording and more extensive analysis of the problem can be achieved as a first step toward the solution.

In most research papers, the SPLs have been recorded over short periods, several days, and even shorter [14,15,16]. In addition to that, the recording of SPLs in some studies has been made only in a one-time shift each day (8–12 h) with a one-hour resolution, i.e., the time interval between the measurements [17]. The analysis of those measurements and recorded sound levels, therefore, could not precisely provide a full picture of the acoustic environment. Very few researchers, however, have measured and analysed sound levels for long periods of time [8]. To efficiently assess the variation of SPLs and study the soundscape in any type of ICU properly and in detail, there is a need for continuous data collection over a longer period of several weeks and even months. The majority of studies have also not tried to establish the severity of the problem over different and distinguishable time shifts.

To overcome the issues encountered in previous studies, the aim of this paper is first to present a study that surveys the literature and reviews some important research papers dealing with this problem. The second aim is to investigate and understand the daily patterns and variations in SPLs recorded over long periods in ICUs in three different hospitals in Spain. The third aim is to examine the similarities and differences in acoustic environment between different periods within the ICU itself, and among other analysed units. To achieve those aims, time and frequency-domain analyses, as well as some statistical approaches, have been performed. Time-domain analysis initially involves transforming the recorded SPLs from a logarithmic scale to a linear scale to see the original pattern of acoustic noise. The recorded SPL time series is then decomposed into three groups to form three distinct periods (three shifts) during the 24 h operation of each ICU—daytime, evening, and night shifts. This helps to illustrate the severity of the problem in different periods during the day. To perform the frequency domain analysis, the discrete Fourier transform (DFT) has been applied, and the cyclic behaviour of the SPLs has been revealed and analysed. By taking the most dominant frequency components and converting them back to the time domain using the inverse of DFT, the background noise (high-frequency SPL variations) can be removed, and the periodicity in the SPL time series is fully exposed. The analysis might help predict the daily acoustic characteristics. This could help in observing the occurrence of high SPLs during specific periods and eventual prevention of those situations, where possible. Finally, statistical analysis has been performed, i.e., average, min, and max SPLs have been established for each ICU, and histograms were obtained to provide a better picture of SPLs to which patients are most often exposed during their stay in ICUs. The histogram shows how many times each SPL occurs over the entire period of recording. Finding the statistical distribution of SPLs provided a suitable means of assessing and comparing acoustic environments through different periods during the day.

The organisation of the paper is as follows: A literature survey is presented in Section 2, which reviews some recent investigations and studies of the problem of noise in ICUs. Details of the data measurement and collection system used in this study are provided in Section 3. Results that include time-frequency analysis and some statistical approaches are presented in Section 4, while findings are discussed in Section 5. Finally, concluding remarks and future work are given in Section 6.

## 2. Literature Surveys and Related Works

Many research papers addressing the problem of existing acoustic and background noise in ICUs from various points of view have been published in recent years. Some studies have proposed passive control strategies to reduce sound levels in ICUs, including changes in materials and building design [18], the use of earplugs or headphones [19,20], and staff education [21]. A summary of some studies and the corresponding passive control techniques used to reduce ICU noise sources is tabulated in Table 2 [17,22,23,24]. A modification in the structural reconstructions was implemented in the study presented by Krueger et al. [17]. The SPLs were recorded for nine days, eight consecutive hours each day before reconstruction, while two days of data were recorded after reconstruction. Although the reconstruction cost was not mentioned, the results reveal a significant decrease in average sound level before and after reconstruction, from 60.44 to 56.4 dBA, respectively. However, there is an increase in the maximum level from 78.39 to 90.6 dBA. The inconsistency in results between the average and maximum sound levels is not explained, and the reductions are still far away from the recommended standard of 45 dBA. Kahn et al. [22] applied a three-week educational programme to modify staff behaviour, raising awareness of the effects of noise pollution. It was found that the number of peaks that have levels ≥80 dBA, reduced from 1363 to 976 after applying the program. Although the result indicates the effectiveness of this technique, the study was carried out over a short period, only two consecutive days, which makes it difficult to generalise the findings of this study. The noise reduction programme proposed by Ford et al. [23] includes daily educational presentations lasting 15 min over two weeks, sound detection for behavioural modification, and low-cost environmental alterations. The authors found no changes in the sound level before and after applying the program; thus, sound-absorbing tiles were recommended at the conclusion. The analysis of sound sources was documented by MacKenzie and Galbrun in [24]. The authors observed the maximum SPLs every minute in three different units for a day (24 h). The study briefly identified 86 noise sources, of which 34% of them can be avoidable and 28% are partially avoidable. However, the duration of the study is very short, and how the noise sources classified was not mentioned.

An analysis of noise sources and related sound pressure levels in the frequency domain has been investigated to help understand the distribution of noise energy in different bands of frequencies. A summary of some studies and the corresponding frequency domain analysis is shown in Table 3 [25,26,27,28]. Vishniac et al. [25] used the octave band filters to reveal that the frequencies between 63 and 1000 Hz (the speech band) had almost constant sound intensity levels. However, the higher frequencies (>1000 Hz) and the low frequencies (<63 Hz) had low and high sound intensities, respectively. Spectral analysis was also performed and proposed by Livera et al. [26] to analyse the noise generated by equipment and activities in the neonatal intensive care unit (NICU) over 15 days, with one measurement each hour at the centre of the NICU room. SPLs, belonging to the spectrum 1–8 kHz, were found to be higher than lower frequencies. Different noise reduction protocols were proposed. However, the low resolution, which is one measurement every hour, is insufficient to provide a full picture of the sound levels in the NICU. Vuksanovic et al. [27] have studied the problem using singular spectrum analysis (SSA) to analyse the data recorded in ICUs over a long period, in which time-series of SPLs can be decomposed into individual components for easier interpretation. Extracting periodic noise from random background noise has been investigated. The authors have also proposed a method based on the SSA to estimate missing measurement data. However, the implemented approach is slightly complex, requiring high execution time in computing software. Konkani et al. [28] applied behaviour modification techniques to reduce noise levels in an ICU based on frequency analysis of recorded data. As revealed in their paper, patient rooms were dominated by high-frequency components with average SPLs of around 69 dB. The nursing station rooms involve both low and high-frequency components. Ultimately, the applied programme was found to be insufficient in reducing noise levels.

Few research studies have addressed the problem using the technique of active noise control (ANC), as illustrated in Table 4. An ANC relies on the phenomenon of incident wave summing. Systems such as ANC were employed first in manufacturing and industries such as venting and engine exhaust systems. Later, this technique was developed for consumer electronics devices, for example, noise cancellation headphones and snore cancellation pillows. Recently, ANC systems have been implemented and developed in medical and healthcare environments [29,30,31]. Hutchinson et al. [29] deployed ANC for the premature infant in a simulated incubator in an NICU. The research experiment was conducted at the Children’s Hospital of San Antonio, Texas. For certain alarm sounds, it was found that the SPLs decreased after applying ANC by 14.4 dB at the alarm tone’s primary frequency. However, the results reveal that the ANC was not able to attenuate SPLs below 40 dB. Further clinical studies are needed, as suggested by the authors, to verify the health benefits. A multichannel feedforward ANC system was also proposed by Congzhi et al. in [30] to reduce the acoustic noise around ICU residents. However, this paper introduced the virtual sensing method, moving to a quiet zone when physical sensors are hard to place at the desired locations. Although simulation results revealed that the proposed technique could reduce sound levels around patients’ ears, the reduction is not as expected compared with the conventional ANC system. The reason behind the poor performance is due to so many impulses in ICU noise, as explained by the authors. Later, Liu et al. [31] proposed an ANC system. They have developed and implemented a multichannel feedforward ANC based on a filtered x least mean square (FxLMS) algorithm using the TMS320 DSP C6713 board. It has been shown that the proposed ANC system can provide a promising solution by reducing SPLs by about 10 dB. Although a real patient bed has been used to test the system with real recorded ICU noise, the system was tested without patients and daily caregiving activities. Further clinical studies are needed.

Many factors can affect the environmental characteristics of ICU wards, including the number of beds, the number and type of medical equipment around patients, the number of nursing staff, the number of patients, the ICU ward’s year of construction, etc. In addition, many factors can affect research findings, as noticed in the research literature, including measurement protocols, duration of the study, types of sound level meters, and measurement resolutions. All these factors make the comparison between research papers difficult to achieve. Therefore, it is unfair and impractical to generalise the problem and its findings.

## 3. Monitoring System, Measurements, and ICU General Environment

**Figure 1:**
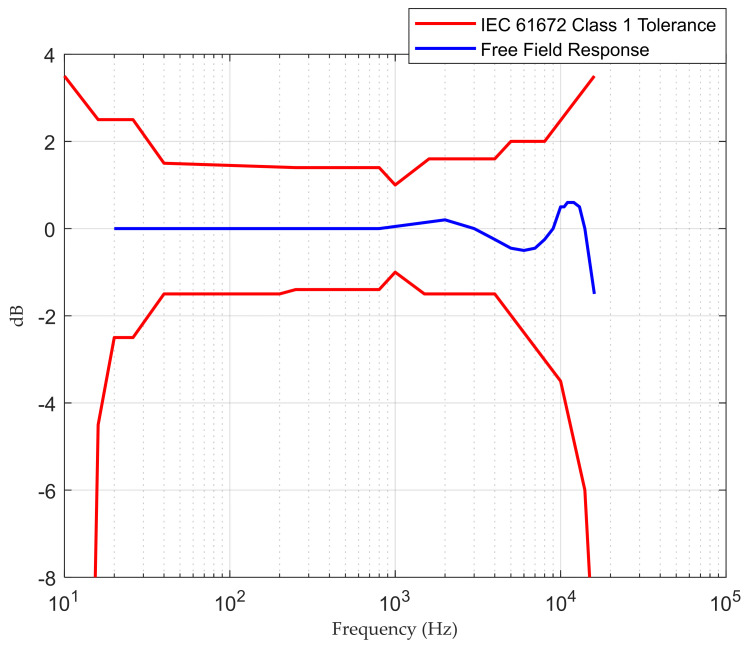
Frequency response of SAS 2000.

Data used in this work have been obtained using a SAS 2000 data logging system (Sound Ambience Supervisor 2000) developed by the EcuDap company located in Burgos, Spain. The system is based around TMS320C6713B digital signal processing, and it uses a 24-bit Delta Sigma ADC/DAC to perform data acquisition. Further technical specifications for SAS 2000 are given in Table 5, while the system frequency response is shown in Figure 1.

The SAS 2000 records, monitors, and visualises SPLs and other physical parameters in the ICU, including temperature, humidity, and luminosity, with one minute of resolution. The system updates the SPL (dBA) value every 15 s and uses the SPLs registered during the previous 60 s for further calculations of subjective parameters, such as loudness. The SPLs are not only measured and recorded locally but are sent and stored via the Internet to a data management platform for further analysis and monitoring. The device uses a display system resembling a traffic light code, that is, red for “too noisy space”, orange for “noisy space”, and green for “comfortable space”.

**Figure 2:**
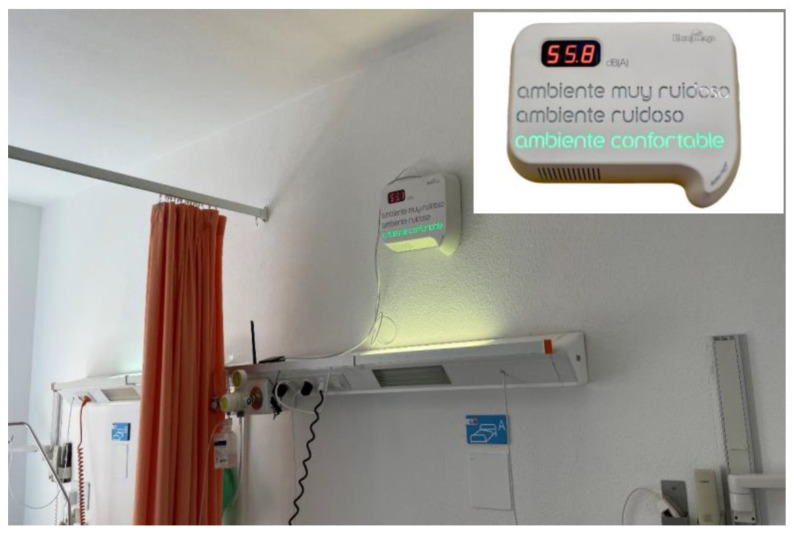
Monitoring system, SAS 2000, in a typical ICU.

SAS 2000 has been installed in several ICUs in hospitals in Northern Spain, and the data sets for this study were collected in three different hospitals. One of the installed devices is shown in Figure 2. The first two data sets were recorded from the period starting at 08:00 a.m. on 1 January 2019 and ending at 08:00 a.m. on 21 January 2019 (20 days). The third data set was collected over the period starting at 08:00 a.m. on 1 February 2019 and ending at 08:00 a.m. on 21 February 2019 (20 full days). Therefore, a total of 28,800 samples at 60-s intervals were recorded at each ICU.

The ICU layout is an open plan with multiple patient beds, each equipped with many different medical devices. The units are also fitted with equipment with significant noise contributions, such as air conditioning, ventilation, alarms, and pagers. Acoustic environments in ICUs are found to be busy with staff and visitors’ conversations as well as caregiving activities such as monitoring patients, recording different data, opening disposable packages, closing doors, storage units, and drawers. Moreover, there are other noise sources, including rolling carts in the corridor, toilet flushes, and vacuum cleaners. The number of staff (nurses, physicians) in ICU wards varies by day and is determined based on the number of patients and their care needs, whereas handovers between shifts are consistent.

## 4. Results of Data Analysis

In this section, the time and frequency domain analyses as well as some statistical approaches of three measured sets of SPLs are presented. All recorded SPLs are in logarithmic measures dB.

### 4.1. Analysis of SPLs in Time and Frequency Domain

**Figure 3:**
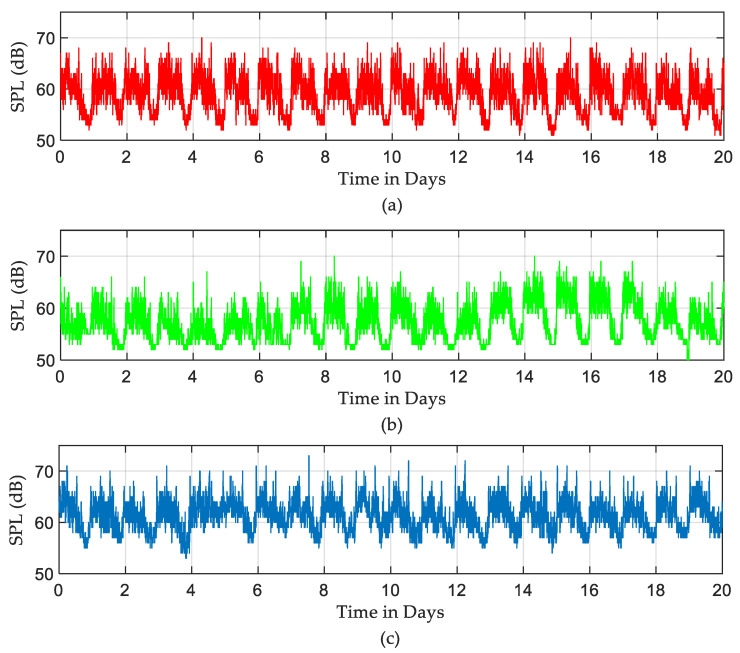
Recorded SPLs in dB over 20 days: (**a**) ICU1, (**b**) ICU2, and (**c**) ICU3.

**Figure 4:**
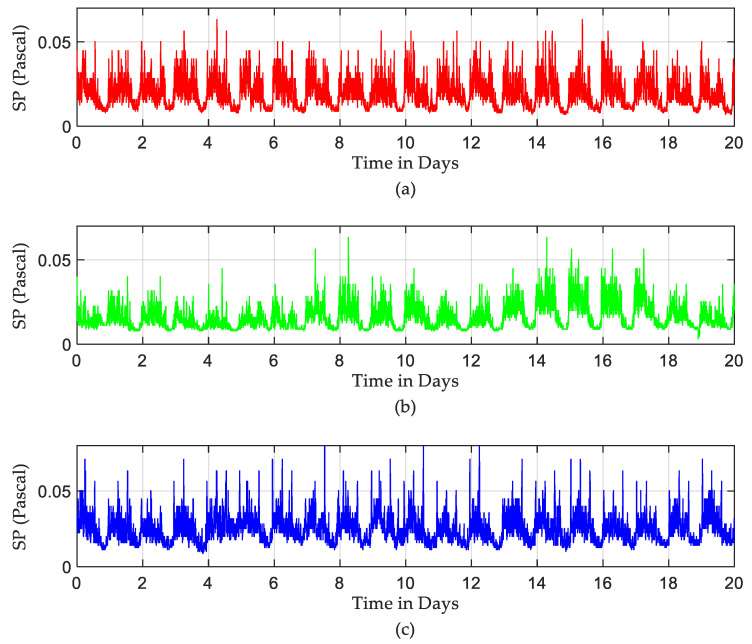
Actual pattens of sound levels in pascal over 20 days: (**a**) ICU1, (**b**) ICU2, and (**c**) ICU3.

Raw recorded SPLs used in this study are shown in Figure 3. Those SPLs were recorded in three different ICUs in the hospitals in Northern Spain over a period of 20 days between January and February 2019. One can note from Figure 3 that SPLs in all ICUs over 20 days exceeded the acceptable levels. Moreover, ICU3 seemed very noisy compared to other ICUs. An anti-log calculation [32] was performed on the recorded SPLs to obtain the actual pattern of sound levels. Those patterns are shown in Figure 4.

**Figure 5:**
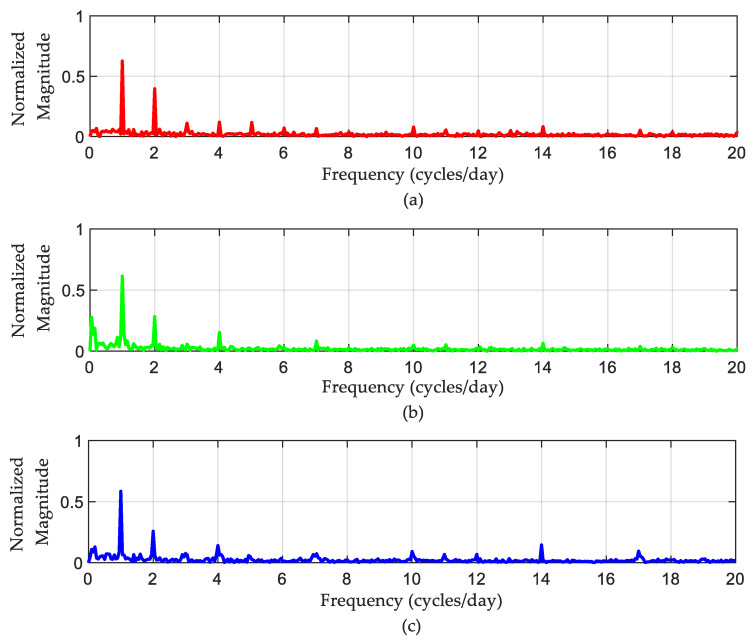
Normalised magnitude spectrum of the recorded SPLs using FFT: (**a**) ICU1, (**b**) ICU2, and (**c**) ICU3.

Figure 5 shows the results of the spectral analysis of those SPL measurements obtained in Figure 4. The magnitudes of the FFT output are plotted against a frequency axis scaled to cycles/day. Dominant frequency components can be observed in the low-frequency region, corresponding to 1, 2, 3, and 4 cycles/day, respectively. Those components can therefore be taken as an indicator of how often noisy events occur during the day. The noisiest events occur once or twice daily, while the events occurring more often are weaker.

**Figure 6:**
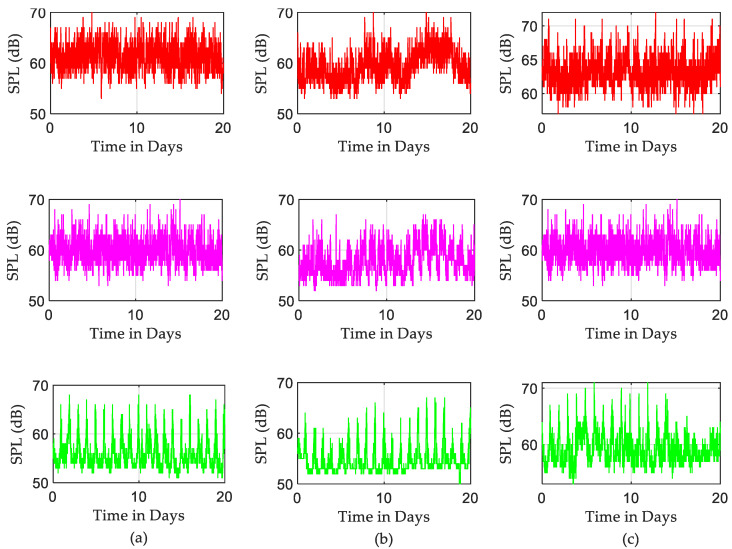
Decomposition the time series of the recorded SPLs; red, pink, and green column plots are SPL time series for daytime, evening, and night (each 8 h a day over 20 days): (**a**) ICU1, (**b**) ICU2, and (**c**) ICU3.

To establish the severity of the problem through different and distinguishable periods during the day, the recorded SPLs are decomposed in the time domain into three groups forming daytime data (8:00 a.m.–4:00 p.m.), evening data (4:00 p.m.–00:00 a.m.), and night data (00:00 a.m.–8:00 a.m.). Each subgroup consists of 8 h of data recording, which includes 480 consecutive samples at 60-s intervals. Figure 6 presents the raw, measured SPLs for each ICU for each of the three periods. It is worth noting that there are significant levels of noise generated during daytime and evening periods. This is most likely due to daily routine schedules such as visitors and regular activities of staff. Peaks at the end of nights and the beginning of morning times can be seen clearly in the corresponding night subplots of Figure 6. This covers the period between 6:00 a.m. to 8:00 a.m. due to the morning shift starting. The third ICU seems significantly busier and noisier during the nights, with some SPLs exceeding 70 dB.

**Figure 7:**
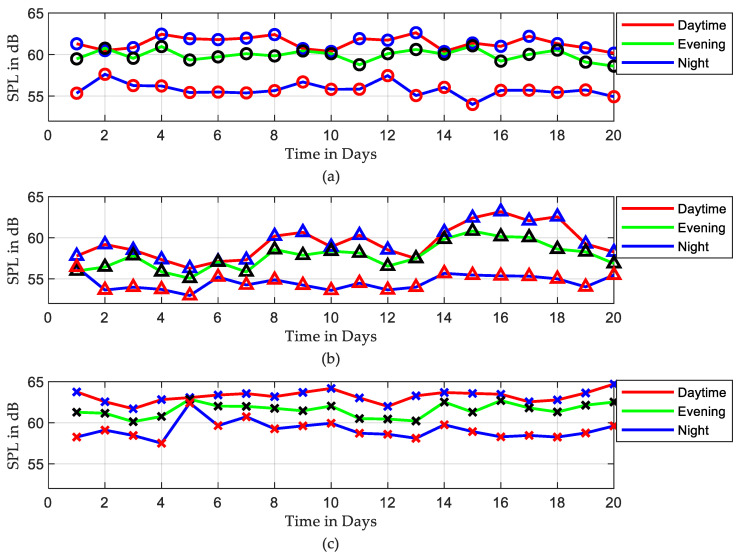
Average of SPLs among three periods over 20 days, the red, green, and blue curves represent the average of SPLs for daytime, evening, and night, respectively, (**a**) ICU1, (**b**) ICU2, and (**c**) ICU3.

Figure 7 depicts the average sound levels over those three periods, where the red, green, and blue curves represent the average sound levels for daytime, evening, and night, respectively. The results show that the daytime period is the noisiest in all three ICUs. Evening times were in second place, with noise levels reaching daytime levels at some points.

**Figure 8:**
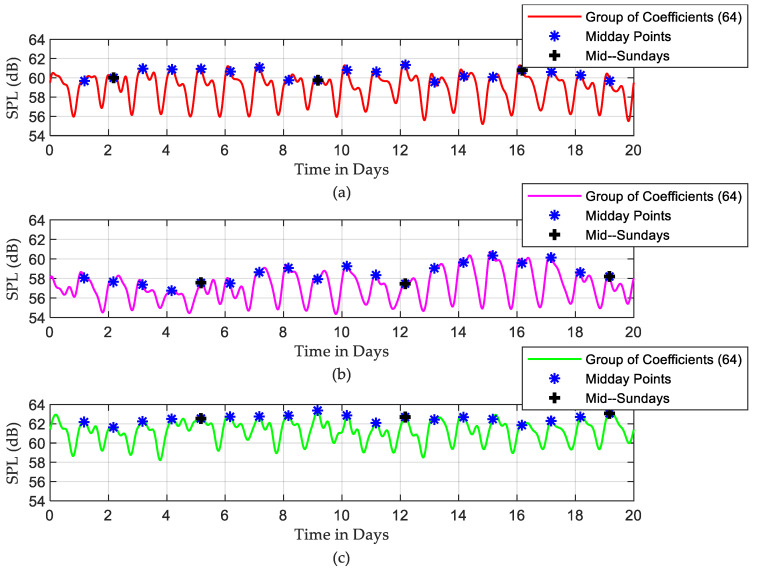
Periodicity of reconstructed SPLs time series over 20 days by employing IFFT to the first 64 points: (**a**) ICU1, (**b**) ICU2, and (**c**) ICU3.

**Figure 9:**
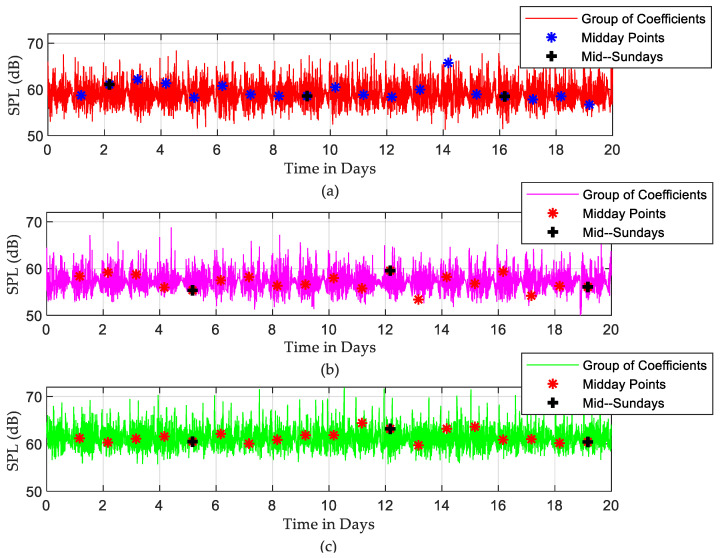
Random noise over 20 days, reconstructed SPL time series by employing IFFT to the rest of the coefficients: (**a**) ICU1, (**b**) ICU2, and (**c**) ICU3.

To analyse the existing periodicities in the recorded SPLs, the determined Fourier decomposition coefficients are grouped, and the inverse of the Fourier transform (IFFT) is performed. The reconstructed SPLs using only the first 64 FFT coefficients are shown in Figure 8. In this way, the periodic noisy events are separated from the random events, and the periodic patterns can be seen more clearly. Midday and mid-Sunday points for each day and weekend, respectively, are also indicated in this plot. The rest of the coefficients are plotted in Figure 9, which mostly shows the random part of the recorded SPL time series. The variances of the random parts are 4.783, 3.408, and 3.963 for ICU1, ICU2, and ICU3, respectively, which represent a very significant contribution to the random noisy events in the recorded SPLs. What is perhaps even more important is that the magnitudes of those random events are generally significantly higher compared to the periodic ones. Midday points (noon) are almost the noisiest times in the periodic signal due to increased activity levels around this period. They consistently coincide with daily peak positions in the periodic pattern but are not surprisingly randomly distributed in the random part of the recorded SPLs. On Saturday and Sunday (weekends), low sound levels were obtained since daily activities in the ICU were reduced due to variations in staffing numbers and visitors.

**Figure 10:**
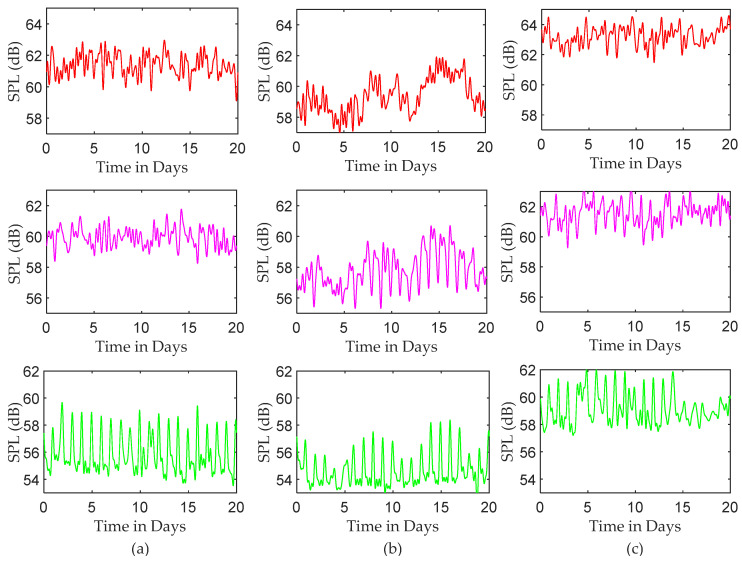
Reconstructed SPL time series for subgroups by employing IFFT to the first 64 points; red, pink, and green column plots are reconstructed SPL time series for daytime, evening, and night (each 8 h a day over 20 days): (**a**) ICU1, (**b**) ICU2, and (**c**) ICU3.

Finally, Figure 10 shows the reconstructed time series of SPLs for three different periods using a similar approach, i.e., the IFFT results when only the first 64 FFT coefficients are used. While each ICU data provides a level of periodicity in the reconstructed time series, most of the periodicity is generated from the sound levels of the night periods. It is obvious, as seen in Figure 10, that the acoustic environments are almost the same during the night.

### 4.2. Statistical Analysis of SPLs

**Figure 11:**
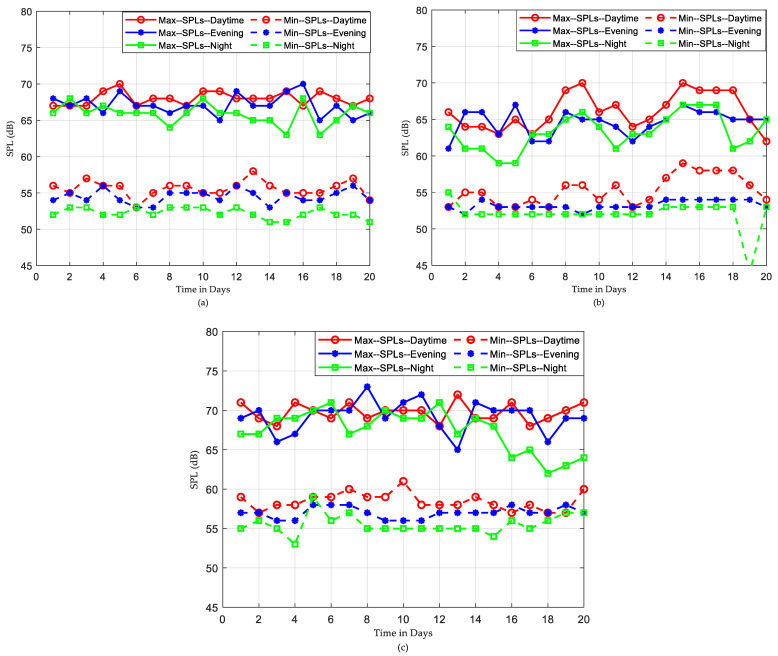
The min and max SPLs during daytime, evening, and night over 20 days for (**a**) ICU1, (**b**) ICU2, and (**c**) ICU3.

This subsection presents a further statistical analysis of the recorded SPLs. The daily maximum and minimum SPL values for subgroups of data are illustrated in Figure 11. It is interesting to note how the max SPL characteristics for the daytime, evening, and night periods are close and similar to each other. The following points regarding the maximum dB values recorded during this period are worth noticing:

- For ICU1, the max SPL curves oscillate between 65–70 dB. Some max points have almost identical dB values, which leads to the conclusion that those common max noise points could be generated from the same acoustic noise sources.
- For ICU2, the max SPL curves abnormally oscillate between 60 and 70 dB. There is an overlap between the max point curves.
- For ICU3, the max SPL curves oscillate between 65 and 73 dB, except for the last five days, as seen in Figure 11c, which show the max SPL curve for the night falls below 65 dB. There is an overlap between the max point curves.

**Figure 12:**
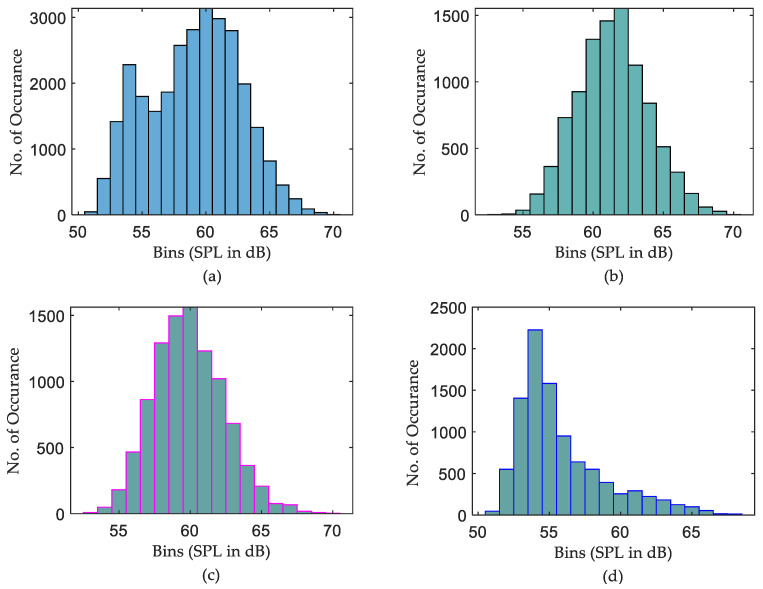
Histogram calculation of ICU1 data over 20 days, (**a**) distribution of SPLs over the entire period, (**b**) distribution of SPLs over daytime, (**c**) distribution of SPLs over evening, and (**d**) distribution of SPLs over night.

**Figure 13:**
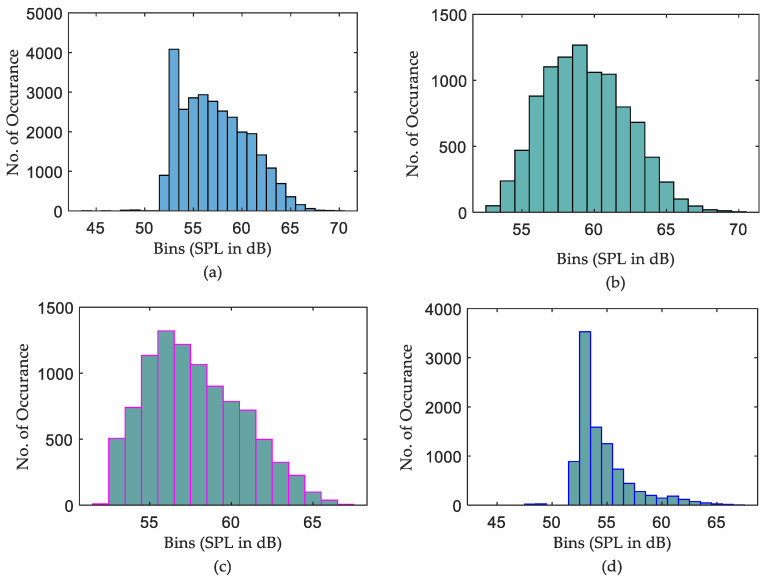
Histogram calculation of ICU2 data over 20 days, (**a**) distribution of SPLs over the entire period, (**b**) distribution of SPLs over daytime, (**c**) distribution of SPLs over evening, and (**d**) distribution of SPLs over night.

**Figure 14:**
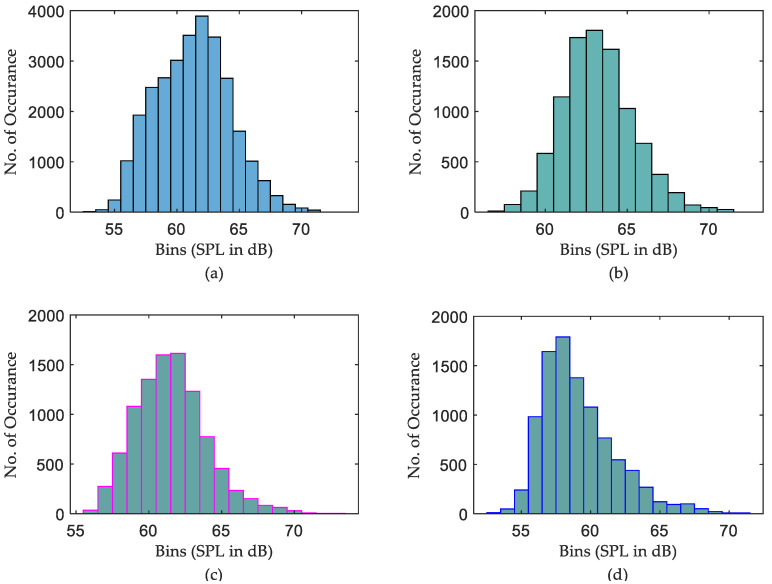
Histogram calculation of ICU3 data over 20 days, (**a**) distribution of SPLs over the entire period, (**b**) distribution of SPLs over daytime, (**c**) distribution of SPLs over evening, and (**d**) distribution of SPLs over night.

On the other hand, the daily min SPL characteristics are much clearer. The min SPLs of night data are slightly lower than those of both daytime and evening data for all ICUs. Nevertheless, it is obvious from Figure 11 that there is no such difference in max curves among the sub-groups on the daily recording over 20 days. This leads to the conclusion that night times are not very quiet periods, with max sound levels often being equal to or greater than the max sound levels for evening periods. However, how many times those max or min measurements occurred during the days is not intelligible. To investigate this issue, a further histogram calculation has been implemented to show how many times exactly each SPL occurs over the entire period of recording. The histograms that illustrate the distribution of SPLs over the entire period, daytime, evening, and night, for the three datasets are depicted in Figure 12, Figure 13 and Figure 14.

It is clear from Figure 12b,c the distribution of SPLs over daytime and evening are almost the same, while a noticeable difference is there if comparing those histograms with the distribution of SPLs over night. Further observations were found that the recorded SPLs over daytime, evening, and night are most of the time between 57–66 dB (95.314% of the daytime readings), 56–65 dB (95.708% of the evening readings), and 52–62 dB (94.458% of the night readings), respectively. However, the distribution of SPLs for each subgroup of data in the second ICU, as seen in Figure 13, is distinct, with almost no similarities between them. Further calculation found that most of the time the SPLs over daytime, evening, and night are between 54–65 dB (97.563% of the daytime readings), 53–64 dB (98.375% of the evening readings), and 52–59 dB (92.864% of the night readings), respectively. Similarly to the second ICU, the distribution of the SPL histogram for each subgroup of data in the third ICU is distinct, as can be seen in Figure 14. In addition to this, it was found that most of the time the SPLs over daytime, evening, and night are between 59–68 dB (97.596% of the daytime readings), 57–66 dB (96.073% of the evening readings), and 55–64 dB (95.178% of the night readings), respectively.

## 5. Discussion

According to the measurements and analysis from the previous section, during the entire period of recording, the SPLs in different ICUs exceeded the recommended levels defined in Table 1. As a simple indication, over 20 days of recording, the SPLs during daytime and evening periods were approximately equal to loud traffic noise. In the early morning shift, as shown in Figure 6, a sudden increase in sound level was identified on each of the monitored days. This is most likely due to regular morning routine activities and interactions between medical personnel and patients. Looking into three defined periods within the 24 h, acoustic scene seems to be very similar for each “Night” period, whereas it can vary significantly during the “Day” or “Evening” periods. One way to show the fluctuation in the recorded data is by finding the average of SPLs. Since there is a significant difference between average sound levels in the three shifts, as seen in Figure 7, there was a significant fluctuation in the recorded data.

The DFT-based analysis extracted the dominant frequency components from the measured SPL time series. According to Figure 5, the noisiest events occur once or twice daily, while the events that happen more often are less noisy. The periodical characteristics of SPL time series were exposed by applying the IDFT to a small number of selected, most dominant DFT coefficients. The purpose of this step is to separate the high-frequency SPL variations from the periodic SPL components, as shown in Figure 8. It was found that mid-day points were the noisiest points in the periodic part of the signal. The DFT approach also depicts the random fluctuation in the recorded data, clearly seen in Figure 9. A significant contribution of the random variations was found in all three ICUs, in which the corresponding variances were found to be 4.783, 3.408, and 3.963, respectively. This finding is consistent with the results achieved by [27], [33], and [34], while it differs from the results achieved by others [1]. However, most of the periodicity was detected during the night, as seen in Figure 10.

It was also found, as shown in Figure 11, that “Night” periods were relatively busy, with max sound levels often being equal to or greater than the max sound levels recorded during “Evening” periods. Constructed histograms have also been useful in understanding which SPL occurs more frequently in different periods. According to Figure 12, for the ICU1, the SPLs occurring more than 1000 times during the three periods over 20 days are 60→63 dB for daytime, 58→62 dB for evening, and 53→55 dB for night, while the most frequent SPLs occurring in the three shifts are 62, 60, and 54, respectively. These statistics can also be constructed for the rest of the data corresponding to Figure 13 and Figure 14. The third ICU seems very noisy around the clock, with some SPLs exceeding 70 dB on some nights.

The question of the underlying causes for each of the high SPLs recorded, however, remains open. This issue will be discussed in more detail in the final section of this paper and addressed in the continuation of this work.

## 6. Conclusions and Future Work

This paper provides a review of the most important and relevant studies, techniques, and attempts used to assess and reduce levels of acoustic noise in ICUs. In the second part of the paper, the problem of noise in ICUs is further illustrated and analysed using data recorded in several ICUs from hospitals in Northern Spain. The presented data have been acquired using commercial equipment performing high-resolution, high-accuracy SPL measurements over extended periods of time. The analysis and new ways of looking into the presented data with a focus on establishing patterns and variations in recorded SPLs have also been suggested.

Different parameters to describe the ICU acoustic environment, such as min, max, and average can be reliably established using a large amount of SPL data recorded over long periods of time, as done in this work. Following this, the decomposition of the recorded SPL time series into three-time shifts was performed which provided further insight into SPL noise patterns, variations, and peak noise periods. The DFT analysis was then applied to investigate the periodicity and the fluctuation present in the SPL time series, which are not easily noticeable in the time domain. However, the real, underlying causes of those variations could not be reliably established from the available SPL data.

Statistical analysis based on a histogram calculation showed the distribution of SPLs over the entire 24 h period, daytime, evening, and night, indicating long exposure of patients to high SPLs. This approach is useful to monitor the soundscape and establish how often each SPL occurs during the entire recording. The findings confirmed the initial notion that the average SPLs in different periods in all monitored ICUs are far from acceptable levels since they exceed the recommended guidelines, indicating an urgent need for their control and reduction.

The approach and results of this work can certainly be useful to medical staff. They help in comparing the acoustic environment between different periods of the day and, even more, between different hospitals and ICUs. Thus, good practice can be shared and common pitfalls avoided. They also help in predicting the daily acoustic characteristics of ICUs and periodic changes, as well as indicating well the random peaks and throughs.

Currently, the authors are trying to address several gaps identified through the presented analysis and discussed in Section 2. It would be of enormous benefit if the underlying causes of the highest SPLs or most sustained periods of high SPLs could be reliably established and identified. This would be a hard or almost impossible task to accomplish using only the SPL recordings performed in most ICUs at the moment, however accurate the recording equipment is. To address this problem, authors are currently developing and deploying a new, intelligent monitoring, IoT-based system to accurately assess the levels and record the sources of acoustic noise in each ICU. Machine learning techniques will then be employed to identify and classify the audio recordings, thus giving reliable information and pointing to the most problematic noise sources and causes. Developed classification and recognition techniques will, in the final stage of this project, be added to the real-time recording system to reliably follow the changes and improvements or, in some cases, the deterioration of the acoustic situation in the ICU, raise the alarm if needed, and point to most persistent “offenders”, i.e., noise sources. The developed system will have significant advantages compared to the pure SPL monitoring described in most of the reviewed literature. What is perhaps even more interesting is that this system should be easily adapted and used for other problematic, noisy environments and spaces. There will certainly be no shortage of candidates in an ever more noise-polluted world of today.

## Figures and Tables

**Table 1:** Standards SPLs in dBA recommended by different organisations.

Time Period	WHO (dBA)	NIC (dBA)	NIOSH (dBA)
Day	35	45	40
Evening	-	40	-
Night	30	20	35

**Table 2:** Summary of the studies and corresponding passive techniques used to reduce ICU noise sources.

Reference	Main Aim	Duration of Study	Method Applied	Conclusions	Some Study Limitations
Krueger et al. (2007) [17]	study sound levels before and after structural reconstruction.	nine days, 8 consecutive hours of recording each day before reconstruction.	modification in structural reconstruction.	significant decreasing in average sound level before and after reconstruction from 60.44 to 56.4 dBA. there is an increase in max level from 78.39 to 90.6 dBA	two days of data collection were recorded after reconstruction. the cost of reconstruction was not noted. the inconsistency in results between average sound level and max level in the study was not explained
Kahn et al. (1998) [22]	identify no. of peaks ≥80 dBA and apply a behavioural modification.	two consecutive days.	three-week educational program to modify staff behaviours.	no. of peaks reduced from 1363 to 976 after applying the program.	the study was carried out over a short period, 2 days. the measurement protocols were not well known.
Taylor-Ford et al. (2008) [23]	proposed noise reduction programs and evaluate the effect of those programs.	six measurement per hour for six days.	daily educational programs for 15 min over two weeks. sound detection for behavioural modification low-cost environmental alterations.	the authors found no changes in the sound level before and after applying the programs. sound-absorbing tiles were recommended at the conclusion.	all the three programs are not well presented. the recording duration interval was not noted. low resolution, six measurements every hour.
MacKenzie et al. (2016) [24]	observe the maximum SPL every minute in three different units.	a single day, one measurement every minute.	analysis of SPLs.	86 noise sources identified in which 34% of them can be avoidable, while 28% of them partially avoidable. talking and rubbish bins were found to contribute more than 10% of noise.	the measurement protocols were not well known. how are sources of noise classified? Based on what? a short period of time, a single day.

**Table 3:** Summary of the studies applied frequency analysis.

Reference	Main Aim	Duration of Study	Method Applied	Conclusions	Some Study Limitations
Vishniac et al. (2005) [25]	analyse SPL in frequency domain.	over years.	use the octave band filters.	the frequencies between 63 and 1000 Hz had almost constant levels. the higher frequencies (>1000 Hz) had low sound intensity levels. low frequencies (<63 Hz) had high sound intensity levels.	the duration of the study is not clear. the measurement protocols were not well known.
Livera et al. (2008) [26]	analyse noise generated by equipment and activities in the neonatal intensive care unit (NICU).	15 days with one measurement each hour.	spectral analysis was performed and proposed.	SPLs, belonging to the spectrum 1–8 kHz, were found to be higher than lower frequencies. sound levels in the NICU are unacceptably high. different noise reduction protocols were suggested.	low resolution, one measurement of SPL every hour. acoustic noise was recorded for one minute, but how often noise segments were recorded is not known. the duration between recordings is not obviously noted.
Vuksanovic et al. (2019) [27]	analyse the data recorded in ICUs over a long period.	34 days, one measurement every minute.	applied singular spectrum analysis (SSA) to discriminate periodic noise from random noise. predict missing data.	the contribution of the random variations in the measured SPL time-series is very significant. the SSA can easily be extended to estimate the missing SPLs.	this study presents data analysis of the SPLs that were measured in a known ICU. complex mathematical approach.
Avinash et al. (2014) [28]	apply a behavioural modification and frequency analysis of recorded data.	7 separate weeks, one measurement each hour.	apply behaviour modification technique using a noise warning device.	patient rooms were dominated by high-frequency components. average SPLs around 69 dB. the applied program found to be insufficient in reducing sound levels.	low resolution, one measurement every hour. very expensive SLM (USD 3345).

**Table 4:** Summary of the studies applied ANC technique.

Reference	Main Aim	Duration of Study	Method Applied	Conclusions	Some Study Limitations
Hutchinson et al. (2020) [29]	proposed an active noise control (ANC) system in a neonatal intensive care unit (NICU).	one time of simulation test.	deployed ANC for a premature infant in a simulated incubator in a neonatal intensive care unit (NICU).	certain alarm sounds, the SPLs were decreased by 14.4 dB. the ANC was more efficient to attenuate frequencies below 2 KHz.	tested for certain alarm sounds. a further clinical study is needed, as suggested by the authors, to verify health benefits. was not able to attenuate SPLs below 40 dB.
Congzhi et al. (2020) [30]	proposed an ANC system to reduce the acoustic noise around ICU residents.	one time of simulation test.	a multichannel feedforward ANC system.	results revealed that the proposed technique can reduce sound level around patient’s ears. the reason behind the poor performance is existed many impulses in ICU noise, as explained by the authors.	the reduction is not as expected as described by the authors. this paper introduced a virtual sensing method and tested without patients. how much SPLs reduced was not mentioned.
Liu et al. (2021) [31]	proposed an ANC system to address high sound levels around ICU patients’ beds.	one time of simulation test.	developed and implemented a multichannel feedforward ANC based on filtered x least mean square (FxLMS) algorithm.	the proposed ANC system can provide a promising solution by reducing sound level about 10 dB.	further clinical study is needed. tested without patients and daily caregiving activities.

**Table 5:** SAS 2000 Technical Data.

Specifications	
Response	Free Field
Diameter	1/4″
Microphone	Integrated
Frequency Response (Hz)	20–20 k
Open-circuit Sensitivity (mV/Pa) (±2 dB)	50
Output Impedance (Ω)	<110
Dynamic Range (dBA)	29–127
Noise (dBA)	29
Operating Temperature (°C)	(−10)–(50)
Operating Humidity (RH)	0–95%

## Data Availability

Not applicable.

## References

[1] Christensen M. (2007). Noise levels in a general intensive care unit: A descriptive study. Nurs. Crit. Care.

[2] Arora R.C., Djaiani G., Rudolph J.L. (2017). Detection, Prevention, and Management of Delirium in the Critically Ill Cardiac Patient and Patients Who Undergo Cardiac Procedures. Can. J. Cardiol..

[3] Van Rompaey B., Elseviers M.M., Van Drom W., Fromont V., Jorens P.G. (2012). The effect of earplugs during the night on the onset of delirium and sleep perception: A randomized controlled trial in intensive care patients. Crit. Care.

[4] Bourne R.S., Mills G. (2004). Sleep disruption in critically ill patients? pharmacological considerations. Anaesthesia.

[5] Thomas K.A., Martin P.A. (2000). NICU sound environment and the potential problems for caregivers. J. Perinatol..

[6] Ryan K.M., Gagnon M., Hanna T., Mello B., Fofana M., Ciottone G., Molloy M. (2016). Noise Pollution: Do We Need a Solution? An Analysis of Noise in a Cardiac Care Unit. Prehospital Disaster Med..

[7] Dias A., Cordeiro R., Corrente J., Gonçalves C.G.D.O. (2006). Association between noise-induced hearing loss and tinnitus. Cad. Saude Publica.

[8] Sayılan A.A., Kulakaç N., Sayılan S. (2021). The effects of noise levels on pain, anxiety, and sleep in patients. Nurs. Crit. Care.

[9] Martellotta F., Della Crociata S., Simone A. Laboratory study on the effects of office noise on mental performance. Proceedings of the Forum Acusticum.

[10] Morrison W.E., Haas E.C., Shaffner D.H., Garrett E.S., Fackler J.C. (2003). Noise, stress, and annoyance in a pediatric intensive care unit. Crit. Care Med..

[11] Ryherd E.E., Waye K.P., Ljungkvist L. (2008). Characterizing noise and perceived work environment in a neurological intensive care unit. J. Acoust. Soc. Am..

[12] Hagerman I., Rasmanis G., Blomkvist V., Ulrich R., Eriksen C.A., Theorell T. (2005). Influence of intensive coronary care acoustics on the quality of care and physiological state of patients. Int. J. Cardiol..

[13] Konkani A., Oakley B. (2012). Noise in hospital intensive care units—A critical review of a critical topic. J. Crit. Care.

[14] Elliott R.M., McKinley S.M., Eager D. (2010). A pilot study of sound levels in an Australian adult general intensive care unit. Noise Health.

[15] Simons K.S., Verweij E., Lemmens P.M.C., Jelfs S., Park M., Spronk P.E., Sonneveld J.P.C., Feijen H.-M., Van Der Steen M.S., Kohlrausch A.G. (2018). Noise in the intensive care unit and its influence on sleep quality: A multicenter observational study in Dutch intensive care units. Crit. Care.

[16] Darbyshire J.L., Young J.D. (2013). An investigation of sound levels on intensive care units with reference to the WHO guidelines. Crit. Care.

[17] Krueger C., Schue S., Parker L. (2007). Neonatal Intensive Care Unit Sound Levels before and after Structural Reconstruction. MCN Am. J. Matern. Nurs..

[18] Berens R.J., Weigle C.G. (1996). Cost analysis of ceiling tile replacement for noise abatement. J. Perinatol..

[19] Mills G.H., Bourne R.S. (2012). Do earplugs stop noise from driving critical care patients into delirium?. Crit. Care.

[20] Richardson A., Allsop M., Coghill E., Turnock C. (2007). Earplugs and eye masks: Do they improve critical care patients’ sleep?. Nurs. Crit. Care.

[21] Moore M.M., Nguyen D., Nolan S.P., Robinson S.P., Ryals B., Imbrie J., Spotnitz W. (1998). Interventions to reduce decibel levels on patient care units. Am. Surg..

[22] Kahn D.M., Cook T.E., Carlisle C.C., Nelson D.L., Kramer N.R., Millman R.P. (1998). Identification and Modification of Environmental Noise in an ICU Setting. Chest.

[23] Taylor-Ford R., Catlin A., LaPlante M., Weinke C. (2008). Effect of a Noise Reduction Program on a Medical—Surgical Unit. Clin. Nurs. Res..

[24] MacKenzie D., Galbrun L. (2016). Noise levels and noise sources in acute care hospital wards. Build. Serv. Eng. Res. Technol..

[25] Busch-Vishniac I.J., West J.E., Barnhill C., Hunter T., Orellana D., Chivukula R. (2005). Noise levels in Johns Hopkins Hospital. J. Acoust. Soc. Am..

[26] Livera M.D., Priya B., Ramesh A., Rao P.N.S., Srilakshmi V., Nagapoornima M., Ramakrishnan A.G., Dominic M. (2008). Swarnarekha Spectral analysis of noise in the neonatal intensive care unit. Indian J. Pediatr..

[27] Vuksanovic B., Arias R., Machimbarrena M., Al-Mosawi M. Monitoring and analysis of noise levels in intensive care units using SSA method. Proceedings of the INTER-NOISE 2019 MADRID—48th International Congress and Exhibition on Noise Control Engineering.

[28] Konkani A., Oakley B., Penprase B. (2014). Reducing Hospital ICU Noise: A Behavior-Based Approach. J. Health Eng..

[29] Hutchinson G., Du L., Ahmad K. (2020). Incubator-based Sound Attenuation: Active Noise Control in A Simulated Clinical Environment. PLoS ONE.

[30] Bi C., Liu L. Multi Channel ANC System with Virtual Sensing Approach for ICU Patient’s Bed. Proceedings of the 2020 IEEE International Conference on Electro Information Technology (EIT).

[31] Liu L., Su Q., Li W., Kuo S.M. Real Time Implementation and Experiments of Multi-channel Active Noise Control System for ICU. Proceedings of the IEEE International Conference on Electro Information Technology.

[32] Švec J.G., Granqvist S. (2018). Tutorial and Guidelines on Measurement of Sound Pressure Level in Voice and Speech. J. Speech Lang. Hear. Res..

[33] Lawson N., Thompson K., Saunders G., Saiz J., Richardson J., Brown D., Ince N., Caldwell M., Pope D. (2010). Sound Intensity and Noise Evaluation in a Critical Care Unit. Am. J. Crit. Care.

[34] Christensen M. (2005). Noise levels in a General Surgical Ward: A descriptive study. J. Clin. Nurs..

